# Do you have COVID-19? An artificial intelligence-based screening tool for COVID-19 using acoustic parametersa)

**DOI:** 10.1121/10.0006104

**Published:** 2021-09-16

**Authors:** Amir Vahedian-azimi, Abdalsamad Keramatfar, Maral Asiaee, Seyed Shahab Atashi, Mandana Nourbakhsh

**Affiliations:** 1 Trauma Research Center, Nursing Faculty, Baqiyatallah University of Medical Sciences, Tehran, Iran; 2 Data Analytics, Scientific Information Database (SID), Tehran, Iran; 3 Department of Linguistics, Faculty of Literature, Alzahra University, Tehran, Iran; 4 Food and Drug Control Department, Jundishapour University of Medical Sciences, Ahvaz, Iran

## Abstract

This study aimed to develop an artificial intelligence (AI)-based tool for screening COVID-19 patients based on the acoustic parameters of their voices. Twenty-five acoustic parameters were extracted from voice samples of 203 COVID-19 patients and 171 healthy individuals who produced a sustained vowel, i.e., /a/, as long as they could after a deep breath. The selected acoustic parameters were from different categories including fundamental frequency and its perturbation, harmonicity, vocal tract function, airflow sufficiency, and periodicity. After the feature extraction, different machine learning methods were tested. A leave-one-subject-out validation scheme was used to tune the hyper-parameters and record the test set results. Then the models were compared based on their accuracy, precision, recall, and F1-score. Based on accuracy (89.71%), recall (91.63%), and F1-score (90.62%), the best model was the feedforward neural network (FFNN). Its precision function (89.63%) was a bit lower than the logistic regression (90.17%). Based on these results and confusion matrices, the FFNN model was employed in the software. This screening tool could be practically used at home and public places to ensure the health of each individual's respiratory system. If there are any related abnormalities in the test taker's voice, the tool recommends that they seek a medical consultant.

## I. INTRODUCTION

Severe acute respiratory syndrome coronavirus 2 (SARS-CoV-2), which causes COVID-19, is a new type of virus that emerged in Wuhan City, Hubei Province in China, in late December 2019 (Lai *et al.*, 2020). Since then, the disease has quickly become a pandemic, killing more than 2.6 million people to date (March 2021) (Worldometer, 2021). COVID-19 is more commonly known as a respiratory illness that transmits through the air and physical contact and penetrates into the respiratory cells by bonding to angiotensin-converting enzyme 2 (ACE2) (Falahi and Kenarkoohi, 2020; Ni *et al.*, 2020). The most common symptoms of the virus include shortness of breath, fever, loss of smell and taste, headache, muscle ache, and cough (Chen *et al.*, 2020; Wang *et al.*, 2020a). The virus is often characterized by specific dysfunction in the respiratory physiology, including the diaphragm and other parts of the lower respiratory tract, thus affecting breathing patterns during inhalation and exhalation of air from the lungs (WHO, 2020). According to many studies, including Osuchowski *et al.* (2021), McGonagle *et al.* (2021), and Gattinoni *et al.* (2020), COVID-19 has a special and distinct pathophysiology from influenza, other non-COVID-19-related acute respiratory distress syndrome (ARDS), and other coronavirus infections (Gattinoni *et al.*, 2020; McGonagle *et al.*, 2021; Osuchowski *et al.*, 2021).

Due to the pandemic, the question “Do I have COVID-19?” often comes to mind after any cough or unusual feeling in the chest or throat. According to the guidelines of the World Health Organization (WHO), nucleic acid-based real time reverse transcription polymerase chain reaction (RT-PCR) is the gold standard method to find COVID-19 positive cases. It is certainly not possible to go to medical centers and perform RT-PCR or chest computed tomographic (CT) scan after every cough or unusual feeling due to the high cost and limited access to the tests as well as the possibility of exposing healthcare professionals and medical staffs to the risk of contracting the virus (Feng *et al.*, 2020; Udugama *et al.*, 2020). Therefore, developing a new accessible and non-invasive approach for screening the disease with higher accuracy is important and can reduce unnecessary worries and prompt the person to take necessary measures.

The respiratory system responsible for providing oxygen to the body is also considered the vocal apparatus's energy generator for phonation production. The exhaled air from lungs maintains subglottalic pressure for the phonatory task. Lung involvement in COVID-19 reduces the pulmonic airflow, and this could cause phonation problems. As reported by Parasher (2021), about one-fifth of all COVID-19 patients have lower respiratory tract involvement that could progress to ARDS.

Speech sound waves are indeed variations in the air pressure produced by the vocal apparatus's different movements (Zhang, 2016). The air stream coming out of the lungs through the trachea provides the necessary energy for speech sounds. The vocal folds located within the larynx may assume different positions, each of which affects the air stream in a different way (Dromey *et al.*, 2002). The laryngeal sound source is created when the adducted vocal folds start vibrating. This laryngeal sound source will then pass through the vocal tract resonators (i.e., all the cavities above the larynx, including the pharynx, mouth, and nose) and is modified and amplified (filtered) by them (Gracco and Lofqvist, 1994). Needless to say, each variation in the shape and function of each organ in the vocal apparatus is reflected in the speech sounds.

Inflammation, edema, or damage to the vocal folds as a cause of infection, coughs, gag syndrome, or the acidity of vomiting materials could affect the function of the larynx, which is directly reflected in the sound waves (Takahashi and Koike, 1976; Oguz *et al.*, 2007; Watts and Awan, 2015; Karlsen *et al.*, 2020). Similarly, tonsillitis and nasal congestion also cause a difference in the shape of the vocal tract that results in variations in the acoustic features of speech sounds. Moreover, an infection or lung injury brings about deviations in the aerodynamic and acoustic features of speech sound waves. Thus, a person with COVID-19 infection not only suffers from shortness of breath, but also has difficulty exhaling, which leads to lack of energy to produce sound and disruption to the speech production cycle as well.

In a recent study, Asiaee *et al.* (2020) reported significant differences in many acoustic parameters, including cepstral peak prominence (CPP), harmonic-to-noise ratio (HNR), the amplitude of the first and second harmonics (H1-H2), fundamental frequency (F0) variation [F0 standard deviation (SD)], maximum phonation time (MPT), and perturbation measures of pitch (jitter) and amplitude (shimmer) between COVID-19 patients and healthy individuals. Quatieri *et al.* (2020) showed that changes in vocal patterns could be a potential biomarker for COVID-19 due to the coordination of subsystems of speech production involving respiration, phonation, and articulation. A study conducted by Bartl-Pokorny *et al.* (2021) compared 88 acoustic features extracted from recordings of the vowels /i:/, /e:/, /o:/, /u:/, and /a:/ produced by 11 symptomatic COVID-19 positive and 11 COVID-19 negative German-speaking participants. Their results revealed significant differences in the mean voiced segment length and the number of voiced segments per second during phonation in COVID-19 positive participants compared to healthy individuals. Since these findings confirmed the significant differences in acoustic parameters of voice between COVID-19 patients and healthy individuals, it is plausible to employ artificial intelligence (AI) as a low-cost, accessible, fast, and non-invasive diagnosis and screening tool to detect COVID-19. These distinctive features can be extracted by appropriate signal processing and mathematical conversion and used to train a sophisticated AI engine to make the initial diagnosis of COVID-19 based on voice quality parameters. AI studies and develops methods that simulate human intelligence (Tayarani, 2021). It reduces the workload required in traditional statistics by screening the data and extracting its important attributes.

Few AI-based studies have been done on the automatic detection of COVID-19, most of which have used x rays (Jain *et al.*, 2020; Ozturk *et al.*, 2020; Wang *et al.*, 2020b; Saha *et al.*, 2021) or CT scans (Harmon *et al.*, 2020; Li *et al.*, 2020). Although these methods (i.e., x rays and CT scans) offer higher sensitivities than the proposed approaches, but both of these methods still require referral to well-equipped clinical centers. A study conducted by Imran *et al.* (2020) used a convolutional neural network (CNN) to introduce a new application to perform direct COVID-19 diagnostics based on cough sounds. Moreover, Mouawad *et al.* (2021) showed the robustness of mel-frequency cepstral coefficient (MFCC) features for automatic detection of COVID-19 through cough and sustained vowel, respectively. A small portion of studies on COVID-19 have focused on machine learning (ML)-based voice quality, a topic that has recently come under the scrutiny of researchers. In a study by Han *et al.* (2020), speech recordings from COVID-19 patients are analyzed to categorize automatically the health state of patients from four aspects, namely, severity of illness, sleep quality, fatigue, and anxiety. In addition, an article by Brown *et al.* (2020) analyzed a large dataset of respiratory resources collected to aid diagnosis of COVID-19. They used coughing and breathing to distinguish between the sounds of COVID-19 from those with asthma or healthy people. The results showed that the classifier was able to classify healthy and COVID-19 sounds using the binary ML classifier and achieved an area under the curve (AUC) of 80% across all tasks. Biswas *et al.* (2020) used cough and vowel /a/ samples to investigate the possibility of using intelligent speech analysis to identify COVID-19 in individuals with and without the coronavirus. Shimon *et al.* (2021) used cough and vowel /a/ samples to investigate the possibility of using intelligent speech analysis for the identification of COVID-19 in individuals with and without the coronavirus. They developed audio-symptomatic models to automatically discriminate between COVID-19 patients and healthy individuals and reported an average of 80% accuracy in detecting the disease based on analyzing coughs and the vowel and 83% accuracy based on the symptomatic questions. In the above-noted studies, different inputs such as sustained vowels, coughing, breathing, and running speech were used. However, in this study, sustained vowel /a/ is used as the input. The rationale behind choosing this type of input is as follows.

Many COVID-19 patients experience respiratory illness (Guan *et al.*, 2020). Respiratory complications of this disease can affect the volume of the lungs and the proper function of vocal cords. During sustained phonation, the F0 perturbation, regularity of vibration, and the harmonicity of sound waves could be measured. However, the glottal abduction, which is followed by a sudden burst in the cough production, makes the measurements of these parameters inaccurate and unreliable. During breathing, there is not any vibration in the vocal folds, so in this case too, the above-noted parameters could not be considered. Meanwhile, lung involvement is another complication caused by COVID-19 that usually results in shortness of breath because of a decrease in the lung volume. Using sustained phonation, the duration of phonation, an important parameter that represents the lung volume, can be studied as well; this cannot be examined in other forms of data including running speech. Moreover, the vowel /a/ is found in most languages of the world, and if a language has a vowel system with either two or three vowels, one of them is /a/ (Maddieson and Disner, 1984; Maddieson and Precoda, 1989; Maddieson *et al.*, 2014). It also has been used as the only sound in many voice-related studies.

In designing an application, it is very important to make it user-friendly. Considering different vowels just makes it difficult and time-consuming for users since there is not much difference in vocal cord function in articulating different vowels. Everybody can use this application from any language whether they are literate or illiterate. Running speech requires individuals to have the ability to read a text in the target language.

In this research, we introduce an AI-based tool that assesses whether any respiratory symptoms related to COVID-19 are detected in an individual's voice solely based on the result of their voice analysis. Therefore, we used the signals of voice to detect COVID-19 and developed a deep learning model that can predict COVID-19. Furthermore, we provided a version of the model as an online service (Vahedian-azimi *et al.*, 2021).

## II. METHODOLOGY

### A. Dataset and subjects

Patients with COVID-19 who were admitted to Baqiyatallah Hospital, Tehran, Iran, from July to September 2020 were enrolled in the study. The diagnosis of COVID-19 infection was confirmed by a positive result in the test (RT-PCR) by a sample collected from nasopharyngeal swab as well as chest CT (WHO, 2020). Therefore, all patients in this study were positive based on two methods. Healthy participants were collected using a simple random sampling method. All participants completed a questionnaire containing demographic characteristics and clinical data. The questionnaire contains questions about demographic data (age, gender, and smoking or addiction history), clinical history [history of asthma, chronic obstructive pulmonary disease (COPD), laryngitis, and chronic bronchitis], and exposure history (history of travel during COVID-19 pandemic and contact with confirmed positive COVID-19 cases). Those healthy individuals who had a traveling history and were exposed to the virus were excluded from the research.

**FIG. 1.**
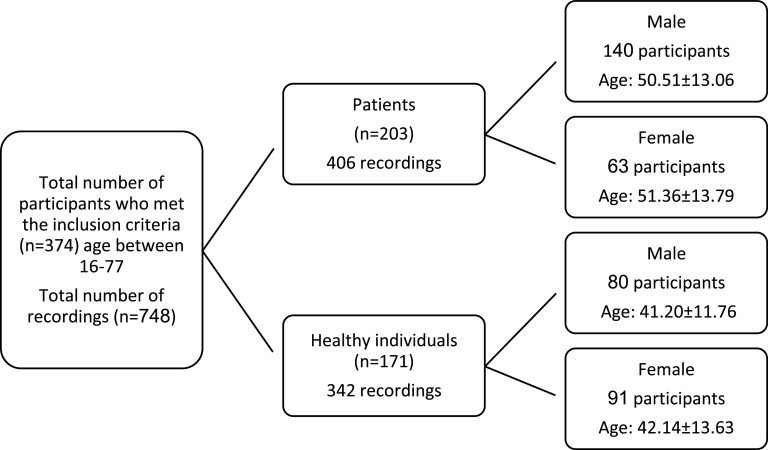
Participants' demographic information.

Two sessions of recordings were carried out per participant. 748 voice samples from both COVID-19 patients and healthy participants were recorded. Samples from healthy participants were recorded in two different sessions. Patients' voices were also recorded in two sessions. The first recording from patients was compiled when they were admitted to the hospital; the second recording was done two days after their hospitalization and when the patients were on medication. Patients' data comprised 406 recordings (280 recordings from 140 male participants and 126 recordings from 63 female participants). Healthy individuals' data contained 342 voice samples (160 samples from 80 male participants and 182 recordings from 91 female participants). Figure 1 shows the participants' demographic information.

The present study obtained the approval of the Ethics Committee of the National Institute for Medical Research Development (NIMAD) for the study protocol under the code IR.NIMAD.REC.1399.056. All the participants had given their informed consent to use their speech samples for research purposes in this observational case-control study.

### B. Voice recording

All recording sessions were conducted using ZOOM H5 handheld recorders with a 44.1 kHz sampling rate and 16-bit quantization. A distance of 20 cm with a 45° angle from the participant's mouth was maintained during each recording. Each participant was asked to take a deep breath and articulate a sustained vowel, videlicet /a/, as long as they could at their comfortable pitch and a constant amplitude.

Two hospital nurses performed the recordings of voice samples from patients. All safety measures, including wearing face masks, face shields, gloves, disposable suits, and sterilizing the recorder with alcohol pads before and after each recording, were observed by the nurses.

### C. Feature definition

Considering the effect of COVID-19 on the vocal organs and its reflection on the acoustic signal of speech sound, we developed a deep multi-layer artificial neural network based on 25 acoustic features and one demographic feature (i.e., sex). These parameters, which are based on different categories, are presented in Table I.

**TABLE I.** Stratification of acoustic parameters implemented in the software based on their different categories.

Category	Parameters
Fundamental frequency (F0)	Mean of F0
	Median of F0
	Minimum F0
	Maximum F0
	SD of F0
F0 perturbation	Jitter local
	Jitter local, absolute
	Jitter rap
	Jitter ppq5
	Jitter ddp
	Shimmer local
	Shimmer local, dB
	Shimmer apq3
	Shimmer apq5
	Shimmer apq11
	Shimmer dda
Harmonicity	Harmonics-to-noise ratio (HNR)
	Noise-to-harmonics ratio (NHR)
	Autocorrelation
	Smoothed CPPs
Vocal tract function	Mel-frequency cepstral coefficients (MFCCs)
Airflow sufficiency	Maximum phonation time (MPT)
Periodicity	Number of voice breaks (NVB)
	Degree of voice breaks (DVB)
	Fraction of locally unvoiced frames

#### 1. F0

F0 is also called the first harmonics (H1) and is defined as the lowest frequency in a complex harmonic soundwave. F0 is directly correlated to the number of times the vocal folds open and close per second. Five parameters of F0 were used in the software, including its mean, median, minimum, maximum, and SD in total duration of phonation. It is assumed that the variability of F0 is increased in COVID-19 patients as the result of damage to the vocal cords.

#### 2. F0 perturbation

It is considered that each cycle's time and amplitude are equal to the next in a harmonic soundwave. However, in the human voice, there are always small variations in the adjacent cycles. Any damage, edema, inflammation, and/or additional mucus in the vocal folds could increase the variability or irregularity in the system. The difference in the duration of adjacent cycles is called jitter. Shimmer, on the other hand, is the difference in the amplitudes of adjacent cycles. Five different formulas of jitter and six formulas for shimmer (Boersma and Weenink, 2020) are implemented in the software.

#### 3. Harmonicity

The vibration of vocal folds is assumed as a harmonic motion producing a complex periodic waveform. If the vibration is aperiodic or the closure phase of vibration is not complete as a cause of vocal fold lesion, a frication noise generates with the turbulence of airflow through the glottis, and the amount of harmonicity decreases. In this software, four parameters (Table I) are considered to measure harmonicity as mentioned in Table I (Boersma, 1993; Hillenbrand and Houde, 1996; Jotz *et al.*, 2002; Hillenbrand, 2011; Teixeira *et al.*, 2013).

#### 4. Vocal tract function

MFCCs are the most common feature used in speech recognition software (Fraile *et al.*, 2009; Godino-Llorente and Gomez-Vilda, 2004). To compute MFCCs, first, the speech signal is split into 20 ms frames, and then discrete Fourier transform is computed from the frames. The mel-frequency warping is the next step that changes the real linear frequency to the logarithmic scale of “mel.” Then a series of triangular filters are used corresponding to the mel-frequency scale, and the center frequency of each filter is defined. As the final step, the mel spectrum is converted to the time domain by performing a discrete cosine transform.

#### 5. Airflow sufficiency

MPT could directly represent air volume in the lungs. It is the total duration of a vowel sound that one can produce after a deep breath (Speyer *et al.*, 2010). The involvement of the lungs in the disease reduces the MPT. Another cause of MPT reduction is inadequate closure of vocal folds and the leakage of air through the glottis.

#### 6. Periodicity

• Number of voice breaks: “The number of distances among consecutive pulses which are longer than 1.25 divided by the pitch floor” (Boersma and Weenink, 2020).
• Degree of voice breaks: “This is the total duration of the breaks among the voiced parts of the signal, divided by the total duration” (Boersma and Weenink, 2020).
• Fraction of locally unvoiced frames: “This is the faction of pitch frames which are analyzed as unvoiced” (Boersma and Weenink, 2020).

### D. Experimental setup

Parselmouth (Jadoul *et al.*, 2018), a python application programming interface (API), was used to access Praat—computer software for the analysis of speech—to extract the features. We standardize the feature values. In effect, we normalize each feature such that the mean will be zero and scale the component to unit variance. No noise removal or signal processing software was used before extracting features. Various ML models, including logistic regression (LR), support vector machine (SVM), k-nearest neighbors (KNN), decision tree (DT), Gaussian naive Bayes (GNB), and feedforward neural network [FFNN, also known as multi-layer perceptron (MLP)] was used. For implementing the ML models, scikit-learn (Pedregosa *et al.*, 2011), a popular ML package, was used. A leave-one-subject-out validation scheme was used to tune the hyper-parameters and record the test set results. The method is essentially done by training the model on all the subjects but one and testing the model on that single subject and repeating the procedure *n* times (where *n* is the number of subjects).

Feature selection is the process of decreasing the number of features when developing a model. It is possible that there is a subset of the features that can be used by the models to improve the performance. To possibly improve the models' performances, we performed univariate feature selection as well. In univariate feature selection, the best features are determined using univariate statistical tests. In effect, we tuned the number of features with other hyperparameters on the validation set. Feature selection was performed to test the possibility of finding better models. Furthermore, we have selected the top *k* feature where 0 < *k* < maximum number of features. The final results are reported based on the leave-one-subject-out method. Table II presents the mean and SD of all parameters sorted by the participants' gender and health status. The last column of the table represents F-score of the univariate statistical tests. The higher the F-score, the more important the parameter is in the model. As can be seen in Table II, the values of MPT, CPPs, and MFCCs are higher than the other features. Meanwhile, features in the category of periodicity, i.e., degree of voice break, fraction of locally unvoiced frames, and number of voice breaks, have the lowest value in the model. Shimmers in general have more values than jitters. Among shimmers, the highest value belongs to shimmer local dB; jitter ppq5 has the highest value among jitters.

**TABLE II.** Descriptive data pertaining to F-score of the univariate statistical tests and acoustic parameters grouped by participants' gender and health status.

	Healthy mean ± SD		Patient mean ± SD		F-score
	Male	Female	Male	Female	
MPT	14.37 ± 6.13	12.40 ± 5.38	6.38 ± 3.60	5.74 ± 2.79	366.82
CPPs	16.78 ± 2.67	14.81 ± 2.33	13.07 ± 3.43	13.42 ± 2.30	139.73
MFCCs	138.14 ± 22.83	109.99 ± 84	146.73 ± 26.61	143.41 ± 22.67	104.42
Shimlocal, dB	0.33 ± 0.20	0.35 ± 0.21	0.64 ± 0.40	0.46 ± 0.33	97.81
Shimapq11	2.88 ± 1.52	2.74 ± 1.72	5.59 ± 3.89	3.50 ± 3.09	86.49
Shimlocal	3.66 ± 2.25	3.77 ± 2.32	6.78 ± 4.56	4.72 ± 3.81	79.66
Shimapq3	1.90 ± 1.26	2.07 ± 1.31	3.52 ± 2.47	2.50 ± 2.08	67.69
HNR	19.71 ± 3.11	20.29 ± 3.56	15.80 ± 5.25	18.87 ± 4.98	68.95
Shimapq3	1.90 ± 1.26	2.07 ± 1.31	3.52 ± 2.47	2.50 ± 2.08	67.69
Shimdda	5.70 ± 3.79	6.22 ± 3.93	10.58 ± 7.40	7.49 ± 6.24	67.69
Jitt ppq5	0.25 ± 0.12	0.26 ± 0.18	0.47 ± 0.50	0.50 ± 0.59	47.32
Auto	0.98 ± 0.02	0.98 ± 0.02	0.93 ± 0.07	0.95 ± 0.06	43.78
Jitter local	0.47 ± 0.26	0.50 ± 0.40	0.92 ± 1.14	1.03 ± 1.33	42.10
Jitt local, abs	3.75E−05	2.620E−05	6.80E−05	5.45E−05	39.62
Jitter rap	0.25 ± 0.16	0.29 ± 0.25	0.51 ± 0.73	0.59 ± 0.84	34.66
Jitter ddp	0.76 ± 0.48	0.88 ± 0.78	1.54 ± 2.18	1.78 ± 2.53	34.65
NHR	0.03 ± 0.03	0.02 ± 0.03	0.10 ± 0.13	0.06 ± 0.09	30.19
Median F0	132.94 ± 24.53	208.16 ± 31.58	141.95 ± 24.93	197.32 ± 30.41	19.63
Mean F0	133.26 ± 24.37	206.70 ± 31.18	142.94 ± 24.91	196.19 ± 27.77	17.16
SD F0	3.71 ± 3.63	9.81 ± 12.67	9.25 ± 12.17	19.85 ± 20.39	16.12
Minimum F0	116.28 ± 19.74	163.90 ± 50.20	123.50 ± 22.25	139.75 ± 41.81	13.30
Maximum F0	164.47 ± 47.89	250.07 ± 50.87	215.94 ± 99.28	263.16 ± 83.51	5.36
DVB	0.43 ± 1.41	0.36 ± 1.07	2.94 ± 9.04	2.57 ± 6.21	2.25
Fraction	1.93 ± 22.83	1.61 ± 1.63	4.13 ± 8.11	4.40 ± 7.05	2.01
NVB	0.41 ± 1.06	0.41 ± 0.82	1.28 ± 2.37	1.17 ± 2.11	1.67

**FIG. 2.**
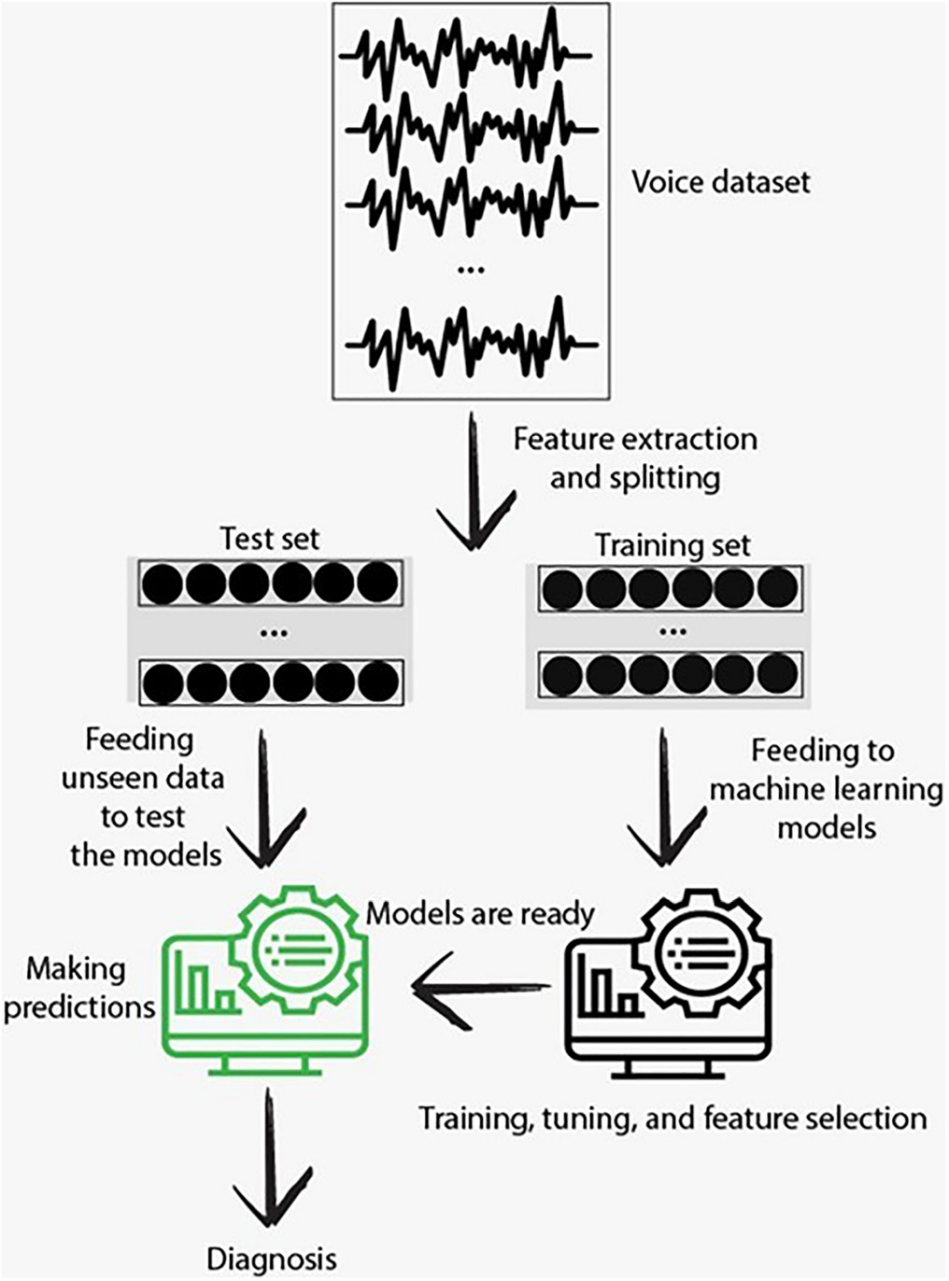
(Color online) Flow chart of data processing.

Figure 2 shows the flow chart of data processing.

## III. RESULTS

The final number of participants whose data were used in the analysis was 374, of which 220 (58.82%) were male. Overall, we had 748 voice samples, of which 406 (54.28%) were for patients and 342 (45.72%) were for healthy individuals. In our dataset, 440 (58.82%) and 308 (41.18%) audio recordings belonged to men and women, respectively.

Information about models' accuracy, precision, recall, and F1-score is presented in Table III. The accuracy, computed based on the total number of true positive (TP) and true negative (TN) divided by the sum of TP, TN, false positive (FP), and false negative (FN), shows the percentage of correct predictions in the test model. The precision refers to the score of TP divided by the sum of TP and FP. Precision delineates the capability of a model to classify a patient as the patient. The recall is calculated based on the ratio of TP to the sum of TP and FN. It represents the classifiers' ability to detect all the samples belonging to patients. In medical situations, if the system incorrectly classifies a healthy person as a patient, it is not as important as when it incorrectly classifies a patient as a healthy person. Therefore, the recall of the patients' class is probably the most important measure when evaluating a model. F1-score is the weighted average of precision and recall. Its preference is for the models that simultaneously have higher precision and recall.

**TABLE III.** Results of accuracy, precision, recall, and F1-score for different ML models based on the total number of true positive (TP), true negative (TN), false positive (FP), and false negative (FN).

Models	Accuracy^a^	Precision^b^	Recall^c^	F1-score^d^
SVM	87.43	86.55	88.18	86.61
LR	89.43	90.17	90.39	90.28
DT	83.02	82.67	86.94	84.75
KNN	85.29	8364	90.64	87.00
FFNN	89.71	89.63	91.63	90.62
GNB	83.29	82.16	88.42	85.18

^a^ Accuracy=(TP+TN)/(TP+TN+FP+FN).

^b^ Precision=TP/(TP+FP).

^c^ Recall=TP/(TP+FN).

^d^ F1−score=[2×(precision×recall)]/(precision+recall).

Based on the data in Table III, representing the models' performances, all models have acceptable performances (more than 80% accuracy). The best results were obtained using FFNN (∼89.71% accuracy) followed by LR (∼89.43%), SVM (87.43%), KNN (85.29%), GNB (83.29%), and DT (83.02%) based on accuracy. FFNN outperforms all the models based on accuracy, recall, and F1-score. LR has the highest precision.

**FIG. 3.**
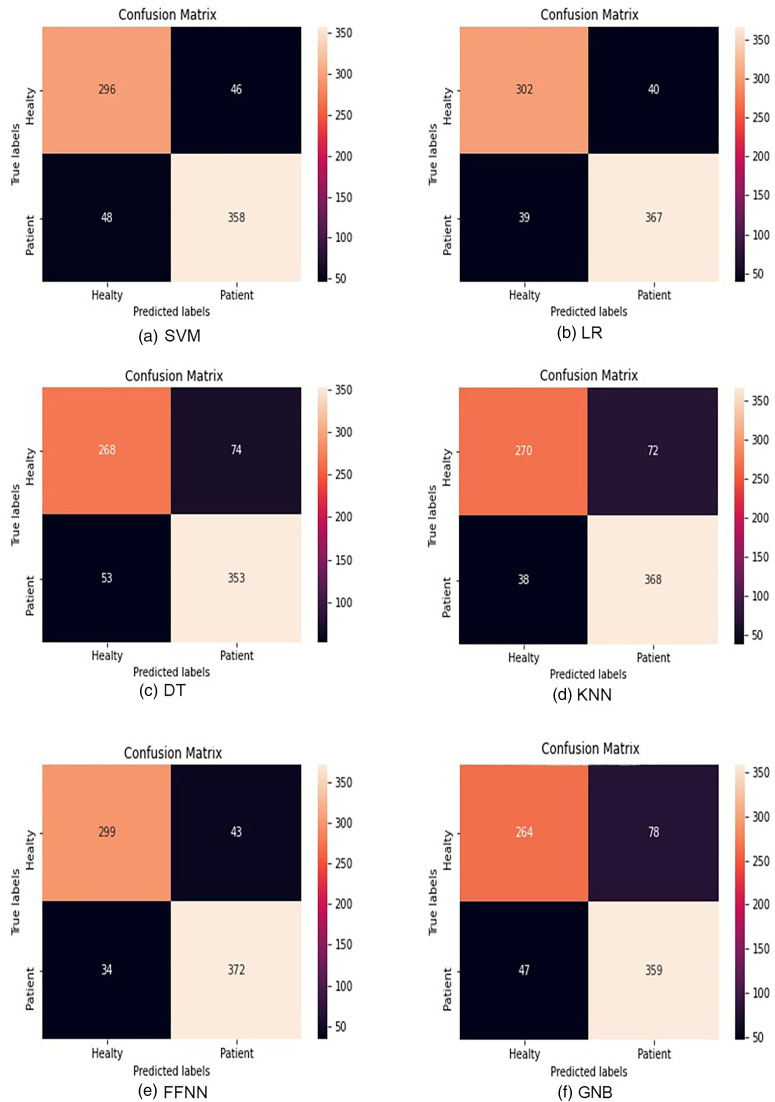
(Color online) Confusion matrices of different models: (a) SVM, (b) LR, (c) DT, (d) KNN, (e) FFNN (MLP), and (f) GNB. The rows of each of the matrices correspond to the ground-truth labels, and the columns illustrate the predicted labels.

Figure 3 delineates the detailed performance evaluation of all investigated models to classify the voice samples, using confusion matrices. The parameters obtained from the elements of the confusion matrix are accuracy, sensitivity, and specificity. These parameters were used for a detailed performance evaluation of the LR, SVM, KNN, DT, FFNN (MLP), and GNB. The rows of each of the confusion matrices correspond to the ground-truth labels, and the columns illustrate the predicted labels. As shown in Fig. 3, the best model, i.e., FFNN, has the best accuracy among all other models and correctly classifies 372 recordings from patients as the patients' recordings from 406 predicted patients' recordings. With its lowest number, FFNN outperforms all other models in mistakenly predicting patients as healthy subjects (lowest FN) as well. This is an important metric for health-related diagnosis. The worst model in this regard is DT, which classifies 53 predicted patients' recordings as healthy ones.

To investigate whether the results are biased or not, the authors performed Spearman's rank-order correlations to determine the relationship between the participant's age and the accuracy of COVID-19 detection for each age. Results showed that the relationship between these two variables was not statistically significant [*r_s_*(34) = 0.051, *p* = 0.769 > 0.05].

Mann–Whitney U test was also run to study the effect of gender on the accuracy of COVID-19 detection. Results showed that the relationship between these two variables was not statistically significant (α > 0.05).

Considering all noted considerations, the FFNN model is chosen as the operating model in the COVID-19 screening tool introduced in the present study. We developed a web application (Vahedian-azimi *et al.*, 2021), which receives the voice of the users and submits the voice to our feature extraction module. After the features are extracted, they are passed to the trained algorithm for detection. The returned value will be passed to the user as the final result. All processes take less than 30 s.

## IV. DISCUSSION

This study was conducted to evaluate the feasibility of using acoustic parameters of voice to screen and diagnose COVID-19 using ML algorithms. Results from studies focused on acoustic parameters of voice in patients with COVID-19 (Asiaee *et al.*, 2020; Brown *et al.*, 2020; Quatieri *et al.*, 2020; Huang *et al.*, 2020) and AI-based surveys confirmed the possibility of employing AI as a tool for screening and detection of COVID-19.

Most of the previous acoustic-based AI tools employed cough, speech, or breathing as their input. The screening tool introduced in this article uses the sustained phonation of a vowel as its input. Vowel phonation has some advantages in comparison to the cough or speech analysis. One of the most important parameters in detecting the disease is MPT, which is directly related to the volume of the lungs. This parameter is absent in cough and speech analysis. Meanwhile, in vowel phonation, we could assess many parameters related to the perturbation, harmonicity, and periodicity of vocal cord vibration. Since there is a strong abduction in the vocal cords and a sudden burst when someone coughs, the analysis of many of these parameters is not possible. Another advantage of vowel analysis in comparison to speech is that it is language-independent.

The unique feature of the present software is using gender as a variable due to the difference between the male and female vocal apparatus.

Unlike conventional diagnostic tests, such as RT-PCR, chest CT, and x rays, this acoustic AI-based tool can be used by anyone anywhere and anytime. Particularly, it is useful for remote areas that do not have access to diagnostic tests, and this tool can act as a clinical decision assistance tool. AI-based tools also provide the chance to better shield physicians from unnecessary exposure to the disease. It also minimizes COVID-19 spread by reducing unnecessary movements and interactions. These tools can also serve as an auxiliary tool that can be used alongside a temperature scanner at airports, borders, or elsewhere as needed.

## V. CONCLUSION

The cost, scarcity, and long duration of clinical tests are the main factors behind the rapid spread of the COVID-19 pandemic. Motivated by the urgent need, this paper presents a preliminary AI-based screening tool for COVID-19 using voice quality parameters. The main idea of this instrument is inspired by the results of our previous study as well as evidence that has shown significant differences in many acoustic parameters between COVID-19 patients and healthy individuals, so these differences can be used as indicators to detect COVID-19 using AI. Using ML algorithms, we proposed and developed a mediator-centered AI-engine for the voice-based diagnosis of COVID-19. The results showed that the service, thanks to the risk prevention architecture, was able to screen COVID-19 with a slight diagnostic error. Despite its impressive performance, it is not meant to be a competitor or a replacement for clinical diagnostic tests. Instead, it is a unique tool for timely, cost-effective, and, most importantly, safe screening, thus controlling the rampant spread of a global pandemic by virtually enabling testing for everyone. This screening tool examines the voice to find respiratory symptoms of COVID-19. For it to be used as a diagnostic tool, it needs to compare voices affected by other respiratory diseases. It should be noted that this is an ongoing project, and the application will be upgraded with more data in the future. A stronger voice-based COVID-19 diagnostic study can be conducted by a larger dataset from other respiratory diseases. Future studies can also increase the number of subjects and the classification accuracy using different feature extraction and classification methods. Moreover, the train and classification stages can be improved by using deep learning algorithms. This work opens the door to further investigation of how automatically analyzed respiratory patterns could be used as pre-screening signals to aid COVID-19 diagnosis.
